# Cytogenetics in *Arctica islandica* (Bivalvia, Arctidae): the Longest Lived Non-Colonial Metazoan

**DOI:** 10.3390/genes9060299

**Published:** 2018-06-13

**Authors:** Daniel García-Souto, Juan J. Pasantes

**Affiliations:** 1 Dpto. Bioquímica, Xenética e Inmunoloxía, Universidade de Vigo, E-36310 Vigo, Spain; danielgarciasouto@gmail.com; 2 CIMUS Biomedical Research Institute, University of Santiago de Compostela, E-15706 Santiago de Compostela, Spain

**Keywords:** ocean quahog, chromosome, fluorescent in situ hybridization, histone genes, ribosomal RNA genes, telomeric sequences

## Abstract

Due to its extraordinary longevity and wide distribution, the ocean quahog *Arctica islandica* has become an important species model in both aging and environmental change research. Notwithstanding that, most genetic studies on ocean quahogs have been focused on fishery related, phylogeographic and phylogenetic aspects but nothing is known about their chromosomes. In this work, the chromosomes of the ocean quahog *Arctica islandica* were analysed by means of 4′,6-diamidino-2-phenylindole (DAPI)/propidium iodide (PI) staining and fluorescent in situ hybridization (FISH) with rDNA, histone gene and telomeric probes. Whilst both 5S rDNA and 45S rDNA were clustered at single subcentromeric locations on the long arms of chromosome pairs 2 and 12, respectively, histone gene clusters located on the short arms of chromosome pairs 7, 10 and 17. As happens with most bivalves, the location of the vertebrate type telomeric sequence clusters was restricted to chromosome ends. The knowledge of the karyotype can facilitate the anchoring of genomic sequences to specific chromosome pairs in this species.

## 1. Introduction

The ocean quahog *Arctica islandica* (Linnaeus, 1767), the longest lived non-colonial animal, is the sole extant species of the family Arcticidae (Bivalvia) and inhabits continental shelves of the North Atlantic [1,2]. As this species has been commercially harvested for decades in North America and Iceland, the better studied aspects of its biology are those more directly related to fisheries and sustainable harvest [3]. The extraordinary longevity of ocean quahogs [1,4] together with its wide distribution have converted *A. islandica* in an important species model in both aging [5,6] and environmental change research [7].

Only a few genetic studies have been performed in ocean quahogs. Mitochondrial cytochrome b gene sequences [8] and random amplified polymorphic DNA [9] were used to evaluate genetic subdivision, demonstrating the existence of genetically distinct, non panmictic populations [9]. Transcriptome libraries were also generated in this species in order to study changes in the expression of oxidative stress related genes [10]. Molecular analysis of telomere dynamics [11] demonstrated that both telomerase activity and telomere lengths were independent of age and habitat in ocean quahogs, therefore suggesting that stable telomere maintenance might contribute to their longevity.

Furthermore, a sequencing project is in progress [12] for the nuclear genome of *Arctica islandica* whereas its mitochondrial genome was already sequenced [13]. As in many other bivalves, doubly uniparental inheritance was detected [14]. The mitochondrial genome sequence of the ocean quahog was used to infer phylogenetic relationships among bivalves [13,15] and the phylogenetic trees recovered branched Arcticoidea either at the basis of Veneroidea [13] or as a sister group of Cyrenoidea and close to Veneroidea [15].

Molecular cytogenetic analyses have been published for a total of 32 species of eight families of heterodont bivalves, Cardiidae [16], Donacidae [17,18,19,20], Mactridae [21,22,23,24], Pharidae [25,26], Psammobidae [27], Veneridae [21,28,29,30,31,32,33,34], Solenidae [35] and Tellinidae [27,36]. Whilst in all of them diploid chromosome numbers were 2n = 38 and vertebrate type hexamere repeats (TTAGGG)n appeared just at telomeric locations, 45S rDNA, 5S rDNAs and H3 histone gene clusters showed differences in their distribution.

Despite the increased interest in the genetics of the ocean quahog, basic genetic data such as chromosome number or karyotype composition are unknown for this species. In this work, we determined the chromosome number and characterized the karyotype of the ocean quahog by staining its chromosomes with 4′,6-diamidino-2-phenylindole (DAPI) and propidium iodide (PI) and fluorescent in situ hybridization (FISH) mapped telomeric sequences and 45S rDNA, 5S rDNAs and H3 histone gene clusters to them.

## 2. Materials and Methods

Bivalve samples were provided by Thalassa Tradition [37]. Ocean quahog handling was conducted in accordance with guidelines and regulations established by the University of Vigo and the local government. The specimens were translated to Toralla Marine Science Station [38], identified according to shell morphology as *Arctica islandica* and maintained in running seawater at 14 °C for one week. Processing of the ocean quahogs was performed following procedures described for other bivalves [39,40]. After overnight exposure to colchicine, specimens were dissected and their sexes determined by microscopically analysing samples of their gonadic tissues. For each ocean quahog adductor muscles were preserved in absolute ethanol and gonadic and gill tissues were immersed in 50% (20 min) and 25% (20 min) sea water, fixed in ethanol:acetic acid (3:1, *v*:*v*, three times, 20 min each) and conserved at −20 °C.

DNA was extracted from adductor muscle tissue with the EZNA Mollusc DNA Kit (Omega Bio-Tek, Norcross, USA) following manufacturer indications. DNA sequences were amplified in a GeneAmp PCR system 9700 (Applied Biosystems, Foster City, USA) [20,23]. The pair of newly designed primers AIS-COIF: 5′TTGAGCAGGATTAATAGGAACT3′ and AIS-COIR: 5′AAATGAACAAATAACACAGGATCT3′ was used to amplify a 630 bp long fragment corresponding to the mitochondrial cytochrome c oxidase subunit I (COI) gene. To confirm the morphological identification of the specimens, the amplified COI gene fragments were sequenced (Supplementary Figure S1).

A nick translation kit (Roche Applied Science, Penzberg, Germany) was used to label 28S rDNA probes with biotin-16-dUTP (Roche Applied Science) or digoxigenin-11-dUTP (10x DIG Labeling Mix, Roche Applied Science). PCR was employed to label H3 histone gene and 5S rDNA probes with biotin-16-dUTP (20 μM) or digoxigenin-11-dUTP (5 μM) [20,23].

To prepare chromosome spreads, pieces of the fixed tissue were disaggregated in 60% acetic acid and the cell suspension spread onto preheated slides [41,42]. After staining with DAPI (0.14 μg/mL) and PI (0.07 μg/mL) for 8 min, chromosome preparations were mounted with antifade (Vectashield, Vector) and photographed using a Nikon Eclipse-800 microscope equipped with a DS-Qi1Mc CCD camera (Nikon, Tokyo, Japan) controlled by the NIS-Elements software (Nikon) [20,23].

FISH mapping of H3 histone, 5S rRNA and 28S rRNA genes was carried out as described previously [43,44]. Karyotype analysis was performed in 10 specimens (5 females, 5 males). At least 10 metaphase plates per specimen were analysed. FISH mapping of the telomeric (C_3_TA_2_)_3_ peptide nucleic acid (PNA) probe (Applied Biosystems) was also performed.

Karyotypes were constructed from 10 metaphase plates obtained from different specimens. Short and long arm lengths were measured for each chromosome. For each metaphase plate, relative lengths of each chromosome pair (100 × chromosome pair length/total length of all chromosome pairs) and centromeric indices of each chromosome (100 × short arm length/total chromosome length) were calculated. Mean relative lengths and centromeric indices were calculated from data obtained from 10 karyotypes. Chromosome nomenclature follows Levan et al. [45].

## 3. Results

**Figure 1:**
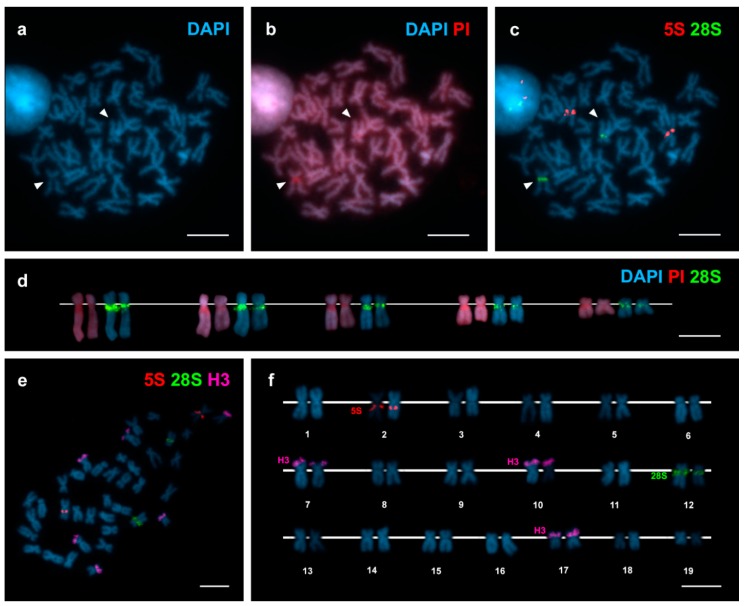
Chromosomal mapping of 28S rDNA, 5S rDNA and H3 histone gene clusters in *Arctica islandica*. Mitotic metaphase plate stained with diamidino-2-phenylindole (DAPI) (**a**) and DAPI/ propidium iodide (PI) (**b**) shows DAPI negative regions (arrowheads) subcentromeric to the long arms of a single chromosome pair; these regions are red after DAPI/PI staining. Hybridization of the same metaphase plate with major and minor rDNA probes labelled differently (**c**) shows major rDNA signals (28S, green) coincident with the DAPI negative regions and minor rDNA signals (5S, red) subcentromeric to the long arms of a metacentric chromosome pair. As shown in (**d**), the sizes of the DAPI negative regions and the 28S rDNA signals, differed between homologous chromosomes irrespective of their condensation degrees. Sequential fluorescent in situ hybridization (FISH) using major and minor rDNA and H3 histone gene probes on the same metaphase plate (**e**) and the corresponding karyotype (**f**), show H3 histone gene signals (H3, magenta) on the short arms of chromosome pairs 7, 10 and 17. Scale bars, 5 μm.

The diploid chromosome number of *Arctica islandica* is 2n = 38 (Figure 1) and its karyotype is composed of 4 metacentric, 1 submeta/metacentric and 14 submetacentric chromosome pairs (Figure 1, Table 1).

DAPI/PI staining (Figure 1a,b) allowed detecting a single 4′,6-diamidino-2-phenylindole (DAPI) negative region close to the centromere on the long arms of a subtelocentric chromosome pair of intermediate size. FISH mapping of 28S rDNA probes demonstrated that major (45S) rDNA clusters mapped to this same position on chromosome pair 12 (Figure 1c,e). The sizes of the DAPI negative regions and the 28S rDNA signals were consistently bigger in one of the two homologous chromosomes (Figure 1d). 5S rDNA clusters also mapped to a single subcentromeric location, on the long arms of metacentric chromosome pair 2 (Figure 1c,e). In contrast, H3 histone gene probes gave signals located on the short arms of three different chromosome pairs 7, 10 and 17 (Figure 1e,f).

**Figure 2:**
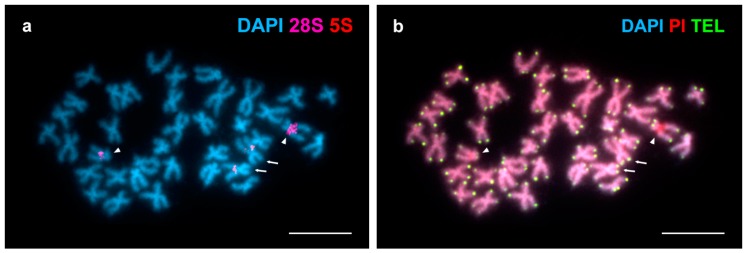
Mapping of telomeric sequences to *Arctica islandica* chromosomes. (**a**) DAPI stained metaphase plate hybridized with major (28S, magenta) and minor (5S, red) rDNA probes. (**b**) The same plate hybridized with a telomeric (TEL) (C_3_TA_2_)_3_ PNA probe (TEL, green) and counterstained with DAPI/PI. Note that the short arms of chromosome pair 2 (arrows) display brighter telomeric signals than their long arms whereas the differences in brightness affect homologous chromosomes in pair 12 (arrowheads). Scale bars, 5 μm.

FISH analysis employing a telomeric (C_3_TA_2_)_3_ PNA probe revealed terminal signals at both ends of every chromosome (Figure 2). The brightness of these telomeric signals showed a certain degree of heterogeneity being some of them brighter than other in homologous chromosomes and even at the chromosome ends of a single chromosome.

## 4. Discussion

To date, chromosome numbers and karyotypes have been described for around 70 species of Heterodonta [23,24,32,33,34,36,46,47,48,49,50]. Although with exceptions as those in Cyrenidae, in which different levels of ploidy have been characterized [51] and Sphaeridae, whose chromosome numbers vary from 28 to 247 [52], most of these species presented diploid chromosome numbers of 2n = 38 and karyotypes showing chromosome pairs with small length differences. As shown in Figure 1 and Table 1, the karyotype of *Arctica islandica* also displayed these characteristics. Furthermore, the absence of telocentric chromosome pairs in *Arctica islandica* is also shared by most heterodont species [26,36].

The occurrence of major rDNA signals in a DAPI negative, GC-rich region in one chromosome pair in *A. islandica* is coincident with the situation in most Heterodonta, in which 24 of the 32 species analysed showed a single location for these clusters [16,17,19,20,22,23,24,25,26,27,28,29,30,31,32,33,34,35]. As in ocean quahogs, nine of these 32 species displayed subcentromeric major rDNA clusters. Heteromorphisms in the size of the DAPI negative regions and the major rDNA FISH signals are also usual in Heterodonta [28,30,32] and are likely the result of differences in the number of copies of the major rDNA repeats in homologous chromosomes.

Regarding 5S rDNA, the occurrence of a single cluster in *Arctica islandica* is shared by another 14 of the 24 heterodont bivalve species previously analysed [16,20,23,24,26,30,31,32,33,34,36] but its subcentromeric location in *Arctica islandica* was previously only described in two other Heterodonta species, the mactra clam *Mactra stultorum* [23] and the tellin shell *Macomangulus tenuis* [36]. In contrast, subcentromeric 5S rDNA clusters were common in marine mussels [53].

In most of the 22 species of Heterodonta studied to date [20,23,24,30,31,32,33,34,36], H3 histone genes were clustered at one (15 species) or two (5 species) locations whereas the remaining two species, the venus clams *Polititapes aureus* [30] and *Chamelea striatula* [34] showed four clusters. The three H3 histone gene clusters detected in ocean quahogs thus represents an unusual situation; this could be related to their subtelomeric location that might facilitate their spreading to non-homologous chromosomes [34].

The detection of vertebrate type telomeric repeats exclusively at chromosome ends in *Arctica islandica* is coincident with results obtained in most bivalves; only the mussel *Perumytilus purpuratus* showed additional intercalary telomeric sequences in two chromosome pairs [41]. Concerning differences in brightness of the telomeric signals in *Arctica islandica*, as variability in telomere fluorescent intensity values within a single metaphase are due to heterogeneity of telomere lengths in mammals [54,55,56,57] probably the same applies to ocean quahogs. If this were the case, it is also possible that the critical element for cell viability in ocean quahogs were not mean telomeric lengths [11] but the lengths of the shortest telomeres [55,56,57]. Telomere quantitative FISH or other methods of telomere measurement [56] need to be applied to address the question.

In summary, as the knowledge of the karyotype of a species, a low-resolution map of its genome, is fundamental in any genome-mapping effort to solve the problems posed by the presence of repetitive sequences and large gene families, the results presented in this study could help anchoring genomic sequences obtained by next-generation sequencing technologies to specific chromosome pairs in the ocean quahog, an important species model in both aging and environmental change research.

## Figures and Tables

**Table 1:** Relative lengths and centromeric indices of *Arctica islandica* chromosomes.

Pair	Relative Length		Centromeric Index		Type *
	Mean	SE	Mean	SE	
1	6.86	0.17	41.35	0.45	m
2	6.12	0.10	36.34	0.74	sm
3	6.05	0.10	42.20	0.67	m
4	5.97	0.13	30.39	4.01	sm
5	5.80	0.12	32.31	3.01	sm
6	5.49	0.08	29.51	1.97	sm
7	5.41	0.12	33.94	1.72	sm
8	5.38	0.09	34.37	1.74	sm
9	5.37	0.08	34.61	2.23	sm
10	5.22	0.11	41.76	0.91	m
11	5.21	0.04	35.47	1.34	sm
12	5.15	0.11	30.07	1.11	sm
13	5.08	0.06	33.39	1.68	sm
14	4.94	0.07	32.09	1.32	sm
15	4.92	0.10	29.50	1.33	sm
16	4.79	0.06	34.37	1.69	sm
17	4.58	0.05	37.10	1.81	sm/m
18	3.94	0.07	33.50	1.01	sm
19	3.71	0.11	43.41	0.89	m

SE: Standard error; * m: metacentric, sm: submetacentric.

## Supplementary Materials

The following are available online at http://www.mdpi.com/2073-4425/9/6/299/s1. Figure S1: Maximum likelihood tree based on mitochondrial COI gene sequences of *Arctica islandica*. References [58,59] are cited in the supplementary materials.
