# Supplementary material for: Cytogenetics in Arctica islandica (Bivalvia, Arctidae): the Longest Lived Non-Colonial Metazoan
Source: Genes (Basel). 2018 Jun 13;9(6):299. doi: 10.3390/genes9060299 (PMC6027238; doi:10.3390/genes9060299)
Supplement: Supplementary file 1 [file genes-09-00299-s001.pdf]

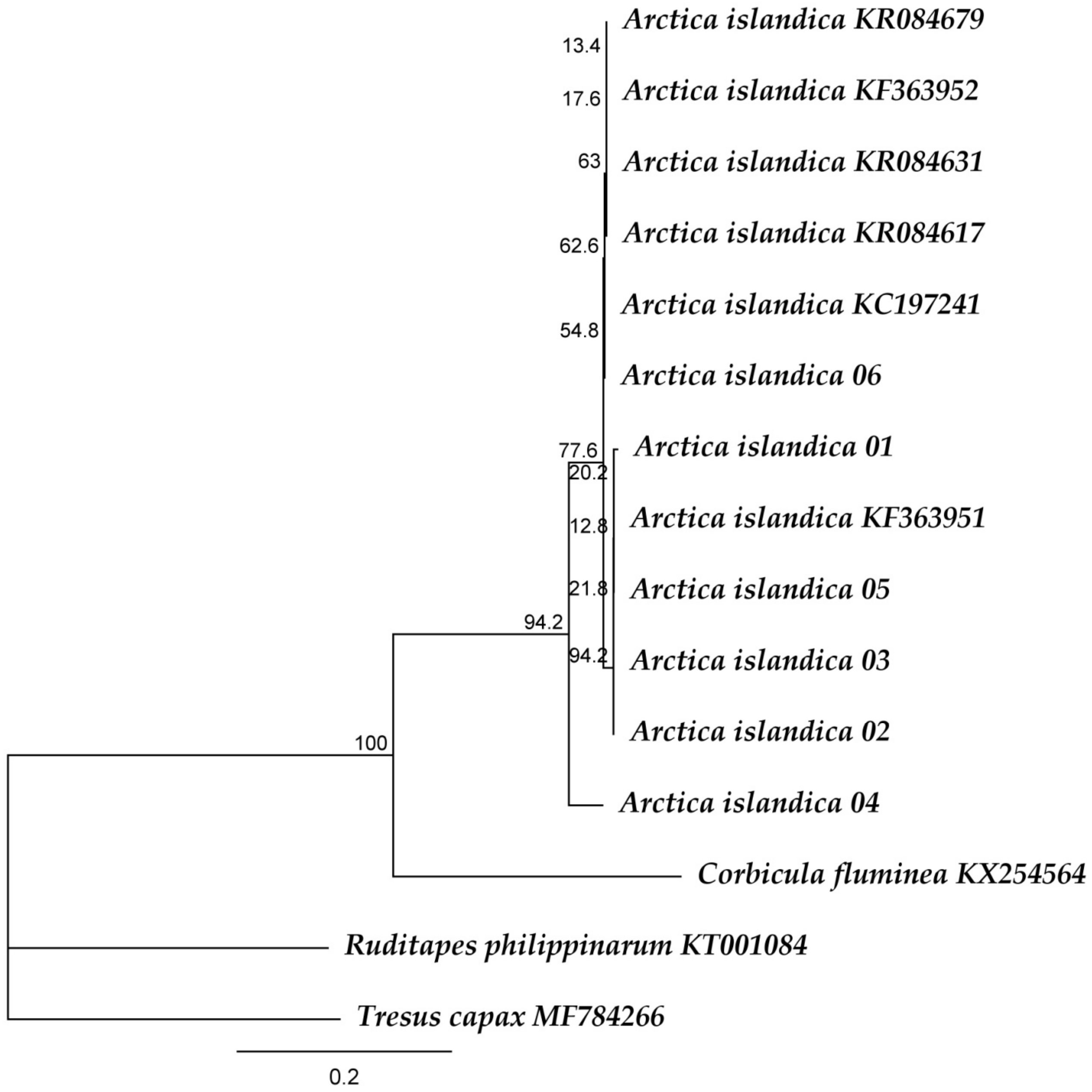

Supplementary Figure 1. Maximum likelihood tree based on mitochondrial COI gene sequences of *Arctica islandica*. *Corbicula fluminea* (Cyrenidae), *Ruditapes philippinarum* (Veneridae) and *Tresux capax* (Mactridae) were used as outgroups. Numbers in internal nodes, reported as percentages, indicate maximum-likelihood bootstrap support values (500 replicates). PCR amplified COI products were purified (FavorPrep™ GEL/PCR Purification Kit, Favorgen) and sequenced (CACTI, University of Vigo) in both directions in an ABI PRISM 3730 Genetic Analyzer (Applied Biosystems) using a BigDye Terminator v3.1 Cycle Sequencing Kit (Applied Biosystems). Geneious V 11.0.4 (http://www.geneious.com) [58] was used to edit and align the sequences. The Basic Local Alignment Search Tool algorithm (BLAST), available at the National Center for Biotechnology Information (NCBI, http://www.ncbi.nlm.nih.gov/blast) was used in sequence similarity searches and the MegaBLAST algorithm set to default parameters was employed against the NCBI nucleotide collection (https://www.ncbi.nlm.nih.gov/nucleotide/) database. COI gene sequences were deposited in the NCBI GenBank database under the accession numbers MH368775 to MH368780. Relationships among sequences were inferred from a maximum likelihood (ML) tree using a TN93+I substitution model. ML reliability was assessed with 500 bootstrap replicates. Analyses were performed with PhyML 3.0 [59] on Geneious V 11.0.4.
